# Molecular Abnormalities Underlying Bone Fragility in Chronic Kidney Disease

**DOI:** 10.1155/2017/3485785

**Published:** 2017-03-22

**Authors:** Yoshiko Iwasaki, Junichiro James Kazama, Masafumi Fukagawa

**Affiliations:** ^1^Department of Health Sciences, Oita University of Nursing and Health Sciences, Oita, Japan; ^2^Department of Nephrology and Hypertension, Fukushima Medical University, Fukushima, Japan; ^3^Division of Nephrology and Metabolism, Tokai University School of Medicine, Kanagawa, Japan

## Abstract

Prevention of bone fractures is one goal of therapy for patients with chronic kidney disease-mineral and bone disorder (CKD-MBD), as indicated by the Kidney Disease: Improving Global Outcomes guidelines. CKD patients, including those on hemodialysis, are at higher risk for fractures and fracture-related death compared to people with normal kidney function. However, few clinicians focus on this issue as it is very difficult to estimate bone fragility. Additionally, uremia-related bone fragility has a more complicated pathological process compared to osteoporosis. There are many uremia-associated factors that contribute to bone fragility, including severe secondary hyperparathyroidism, skeletal resistance to parathyroid hormone, and bone mineralization disorders. Uremia also aggravates bone volume loss, disarranges microarchitecture, and increases the deterioration of material properties of bone through abnormal bone cells or excess oxidative stress. In this review, we outline the prevalence of fractures, the interaction of CKD-MBD with osteoporosis in CKD patients, and discuss possible factors that exacerbate the mechanical properties of bone.

## 1. Introduction

Elderly people are susceptible to diseases such as hypertension, diabetes mellitus, and chronic obstructive pulmonary disease. Osteoporosis and chronic kidney disease (CKD) are also common, and the prevalence of these diseases is increasing globally, in part due to the increasing aging population. Osteoporosis under uremic conditions and management of the disease have not been widely studied. The prevalence and risk of fractures are higher in CKD patients compared to healthy people. Patients on dialysis, in particular, have an approximately fourfold greater risk for hip fractures than sex- and age-matched individuals in the general population [1, 2]. Their fracture risk correlates positively with age, duration of dialysis, high or low parathyroid hormone (PTH) level, female gender, low body mass index, and presence of peripheral vascular calcification. Several studies report that nondialysis patients aged over 50 with estimated glomerular filtration rate (eGFR) below 60 mL/min/1.73 m^2^ also have a twofold greater risk for hip fractures than individuals without CKD [3–7]. A hip fracture critically limits activities of daily living and increases fracture-related mortality [8–10], and this trend is more evident in dialysis patients [11, 12]. Japanese dialysis patients, however, have relatively better prognosis with regard to survival after a hip fracture [13]. A tool called FRAX® that can predict fracture risk appears to be useful for predicting death among Japanese hemodialysis patients [14]. Even though it remains unclear why FRAX was useful to predict mortality in Japanese dialysis patients, elucidation of the pathogenesis of decreased bone strength and treatment of fractures in patients with CKD are important to improve survival and the quality of life in this patient population. In this review, we describe the current status of fragility fractures and their treatments in CKD patients.

## 2. Risk Factors of Fragility Fractures in CKD Patients

Clinicians and researchers agree that risk factors for fractures in CKD are complicated because patients have many abnormalities that may increase fracture incidence. Advanced muscle weakness [15], frailty [16], and deteriorated cognitive function [17] are potential contributors to increased risk for falling among CKD patients. Falls are especially common in older CKD patients [18]. Lack of exposure to sunlight, which contributes to muscle strength, may be a risk factor, because the risk for hip fractures tends to be higher in high-latitude regions of the United States [19]. Despite the high prevalence of hip fractures, clinical studies have failed to elucidate why falling affects the risk for hip fractures but not fractures of other parts of the body such as vertebrae and wrist. In addition, CKD patients also have deteriorated mineral metabolism.

## 3. Definitions of CKD-MBD, Renal Osteodystrophy, and Osteoporosis

The three key bone lesions accompanying CKD are CKD-mineral bone disorder (CKD-MBD), renal osteodystrophy, and osteoporosis, but their definitions are often ambiguous. CKD-MBD is a syndrome defined by the Kidney Disease: Improving Global Outcomes (KDIGO) guidelines as a systemic mineral metabolic disorder associated with CKD, which could result in disorders of bone metabolism and/or the cardiovascular system [20]. CKD-MBD consists of three components; abnormalities of calcium, phosphorus, PTH, and vitamin D metabolism; abnormalities in bone turnover, mineralization, volume, and strength; and soft tissue calcification including vascular calcification. This disease may manifest one component or any combination of the three. According to this definition, “renal osteodystrophy” indicates bone morphologic changes in patients with CKD and is one measure of the skeletal disorder component of CKD-MBD.

Bone lesions accompanying renal dysfunction are symptoms of CKD-MBD, but worsening of mechanical bone strength is not typically mentioned. Impairment of mechanical properties of bone comes under the term “osteoporosis,” as defined by the National Institute of Health. This pathophysiology is characterized by compromised bone strength predisposing a person to increased risk of fractures [21]. In this definition, bone strength is a composite of bone mass and bone quality. Bone mass is a strong determinant of bone strength and is useful as a diagnostic tool for osteoporosis in people with extremely low bone mass. As there are no other tools to predict and/or monitor bone strength in clinical practice, bone mass measurement is considered the most informative and useful tool available to diagnosis osteoporosis. Bone mass, however, is not the only determining factor. Other factors affecting bone mechanical strength include “bone quality.” Bone quality is used to describe the ability of bone to perform mechanical load-bearing functions. This definition includes all characteristics that influence the load-bearing capacity, including bone microarchitecture and material properties [22, 23], Table 1.

A question often arises as to which plays a more important role in bone mechanical strength, bone mass or bone quality. However, the contribution of each of the two parameters remains unclear, because several cohort studies suggest that one-half of all fragility fractures are observed in postmenopausal women with a* T*-score above −2.5 SD, the threshold for diagnosing osteoporosis defined by the World Health Organization [24–26]. Additionally, postmenopausal women with fragility fractures had poor bone microarchitecture and altered material properties, which influence bone mechanical properties [27–29]. Therefore, bone mass measurement is strictly not the standard method for diagnosing osteoporosis.

With the progression of renal function impairment, fracture risk is remarkably high in CKD. While we suspect that osteoporosis may underlie the increased risk of fracture in CKD, the mechanism may differ from that of primary osteoporosis characterized by marked reduction in bone mass. It is also unclear whether osteoporosis (bone fragility) associated with CKD is derived from CKD-MBD or factors other than CKD-MBD.

## 4. Possible Factors Related to Weakening of Bone Strength

Both clinical and preclinical studies suggest that loss of bone strength in CKD patients has two possible components, loss of bone mass and deterioration of bone quality. The KDIGO guidelines published in 2009 do not recommend routine bone mineral density (BMD) testing because BMD does not predict fracture risk in patients with kidney disease as it does in the general population [30]. However, a recent meta-analysis reveals that BMD is significantly lower in predialysis patients with fracture compared to those without [31]. A prospective study has shown that BMD measured by dual X-ray absorptiometry (DXA) predicts incident fracture in stages 3–5 CKD patients, and the prediction ability is comparable to that using high-resolution peripheral quantitative computed tomography [32]. Furthermore, two studies have reported the assessment of BMD using DXA to predict fractures in CKD patients including those on hemodialysis [33, 34]. Therefore, BMD measured by DXA may be useful to assess loss of bone mass or fracture risk. On the other hand, cortical bone loss that increases in advanced stage of CKD is not well depicted by DXA. Therefore, DXA still has limited clinical utility in advanced stage of CKD. More attention should be paid to other factors affecting bone strength. Factors contributing to bone strength comprising bone loss and bone quality are discussed below and summarized in Table 2.

## 5. Humoral Factors Related to Mineral Metabolism

Progressive changes in serum biochemical parameters such as phosphorus, PTH, 1,25(OH)_2_ vitamin D_3_, and fibroblast growth factor 23 (FGF23) levels indicate CKD-related disturbances of mineral and endocrine factors [35, 36]. Increased PTH levels powerfully impact bone mechanical properties, because PTH modifies the activities of bone cells, which regulate bone turnover leading to altered bone mass. PTH stimulates the osteoclastic resorption and remodeling speed, thereby increasing bone turnover. Reduction in cortical BMD and thickness together with increase in cortical porosity assessed by DXA or high-resolution peripheral quantitated tomography (HR-pQCT) have been reported to result in increased bone fragility [37–40]. In stable dialysis patients, Kazama et al. [41] showed that circulating PTH level correlates inversely with cortical porosity but not with cancellous bone volume assessed by bone histomorphometry. Parathyroidectomy in patients on maintenance hemodialysis reduced fracture risk [42]. Additionally, elevated serum alkaline phosphatase due to excessive PTH secretion is associated with higher risk of hip fracture [43]. However, contradicting results on the relationship between PTH level and structure-related bone strength have also been reported [44, 45]. Moreover, medical and surgical treatments for severe hyperactive parathyroid function have progressed, and moderate hyperparathyroidism is unlikely to be a major risk factor for skeletal fragility.

Disturbed bone remodeling (marked decreases in both bone resorption and bone formation) caused by suppressed PTH secretion or skeletal resistance to the action of PTH under uremic condition exits in low-turnover bone lesions in CKD [46–48]. This condition is called “adynamic bone,” and is an increasingly common occurrence [49, 50]. Adynamic bone constitutes 50% of all CKD-MBD in patients on peritoneal dialysis and 19% in patients on hemodialysis [30]. Several clinical and animal studies have suggested an increased fracture risk in adynamic bone disease [51–54].

To summarize, the relationship between fracture risk and PTH level, which alters bone remodeling and bone microstructure, remains controversial. Regardless of high or low PTH level, it is currently difficult to predict fracture risk by PTH level.

FGF23 is derived from osteocytes and is an endocrine hormone that regulates phosphate metabolism. FGF23 stimulates urinary phosphate excretion, suppresses absorption in the gut, and accelerates degradation of 1,25(OH)_2_ vitamin D_3_ in response to a high phosphate diet or a state of impaired phosphate excretion as seen in CKD. FGF23 level is elevated prior to changes in phosphate, 1,25(OH)_2_ vitamin D_3_, and PTH levels accompanying decline in GFR [55–57]. While some studies reported an association between elevated FGF23 secondary to early CKD and risk of fracture in elderly men with decreased eGFR [58–60], other reports found no significant relationship [61, 62]. Isakova et al. [63] analyzed 2234 subjects and reported that FGF23 level was not associated with bone loss or fracture risk in a community-based population of well-functioning older adults. A recent report found that elevated FGF23 induced by high phosphorus diet increased the expression of secreted frizzled-related protein 4 and Diccopf-1, which are Wnt signal inhibitors, and inhibited the Wnt signal pathway [64]. Another report showed that FGF23 also had a physiological role in local bone mineralization, regulating osteopontin indirectly through transcriptional control of tissue nonspecific alkaline phosphatase in a vitamin D- and klotho-independent manner [65]. These reports suggest that high FGF23 level may affect bone fragility by decreasing mineralization through inhibition of the Wnt pathway.

Sclerostin is a Wnt pathway inhibitor secreted by osteocytes. The canonical Wnt/*β*-catenin signaling pathway directly affects osteoblast differentiation, proliferation, survival, and bone formation. Sclerostin antagonizes Wnt signaling and inactivates the pathway. The relationship between sclerostin and fracture risk is not consistent among studies [66–69]. Serum sclerostin is high in early CKD and is maintained at a high level in the advanced stages [70–73]. In an animal study, higher serum phosphate concentration derived from a high phosphorus diet was found to elevate sclerostin expression despite increased osteocyte apoptosis [74]. Combination therapy of anti-sclerostin antibody with PTH-suppressive agent was effective in improving bone mass and mechanical properties [75]. It is possible that a high sclerostin level is an aggravating factor of bone fragility.

Vitamin D [25(OH)D_3_ and 1,25(OH)_2_D_3_] deficiency and altered vitamin D metabolism occur in CKD patients. Because vitamin D is required for normal bone formation and mineralization, 25(OH)D_3_ deficiency (<15 nmoL) is associated with less bone formation and mineralization in trabecular bone [76]. A lower vitamin D status is associated with increased fracture incidence and risk [77–80]. Recently, Murali et al. [65] showed that 1,25(OH)_2_D_3_ inhibits local mineralization by augmenting the expression of the inhibitor osteopontin. To elucidate the involvement of 1,25(OH)_2_D_3_ in bone mineralization in CKD, further in vivo and in vitro experiments are required.

An increased incidence of bone fragility was observed in CKD irrespective of variations in PTH, 1,25(OH)_2_D_3_, FGF23, and sclerostin levels that reflect disturbances of mineral and endocrine metabolism. Factors other than CKD-MBD which may aggravate weakening of bone mechanical properties in CKD patients should be considered.

## 6. Uremic Conditions Deteriorate Bone Material Properties

Bone is composed of two organic materials, type I collagen and hydroxyapatite. The number of collagen crosslinks formed by both enzyme-induced and non-enzyme-induced processes as well as tissue mineral content (density) confer bone elasticity and strength. Various abnormalities in bone material properties are found in CKD patients.

The chemical composition of bone can be analyzed by vibrational spectroscopic methods such as Fourier transform infrared or Raman spectroscopy [81, 82]. These methods provide data on mineral parameters including the mineral-to-matrix ratio (indicating the degree of mineral apposition), the degree of carbonate substituting for phosphate in the apatite lattice, and crystallinity (representing the mineral crystal size and perfection). Additionally, collagen maturity can be obtained by calculating the ratio of mature crosslinks to immature crosslinks. Alterations of these parameters in the bones have been reported in animal models of CKD [51, 83–85] and bone biopsy samples from hemodialysis patients [86, 87].

Nonphysiological collagen crosslinks formed by the actions of advanced glycation end-products are modified crosslinks and are found in increased numbers in bone biopsy samples from dialysis patients [88] and animal models of CKD [51, 83–85]. Immunostaining analysis of bones in a rat model of CKD also demonstrated increase in crosslinks modified by advanced glycation end-products and reduced lysyl oxidase protein, an enzyme required for generating physiological collagen crosslinks [89]. The degree of biological bone apatite orientation, which is related to bone elasticity, was assessed by X-ray diffraction [90] and was found to be exacerbated in a rat model of early kidney injury [84]. Interestingly, in experimental uremic animals, these changes were complicated by the progression of renal dysfunction [51], and some changes were independent of bone turnover [84]. The changes were reduced by administration of AST-120, an oral charcoal adsorbent of uremic toxins [83]. AST-120 did not change mineral metabolism. Therefore, the uremic condition may modify the material properties directly.

Uremic conditions are known to create an excess oxidative stress environment [91]. Uremic condition or a specific uremic toxin inhibits osteoblasts [92–96] and osteoclasts [97]. Although whether the material properties are altered in CKD patients with fragile bone has not been confirmed, uremia-related osteoporosis causing bone fragility should exist in CKD.

## 7. Microcracks and Osteocyte Apoptosis

Because one of the purposes of bone remodeling is to repair microdamage that occurs in bone from daily mechanical stress, suppression or absence of remodeling will result in accumulation of microdamage. Excessive accumulation of microdamage can cause fragility fractures. Although there are no reports that indicate impaired microdamage repair in low-turnover bone associated with CKD, findings that suppressed bone turnover increases fragility and fracture risk suggest accumulation of microdamage [98–100].

The rates of osteocyte apoptosis and reduced density are higher in fractured bone than in normal bone [101–103]. Empty lacunae (absence of osteocytes in lacunae) are found in renal osteodystrophy. PTH fragment, especially the c-terminal PTH fragment, increases osteocyte apoptosis [104]. The c-terminal PTH fragment is accumulated in CKD, and the amount increases depending on renal insufficiency [105]. From these findings, increased osteocyte apoptosis appears to be associated with fragility fractures in CKD patients.

**Figure 1:**
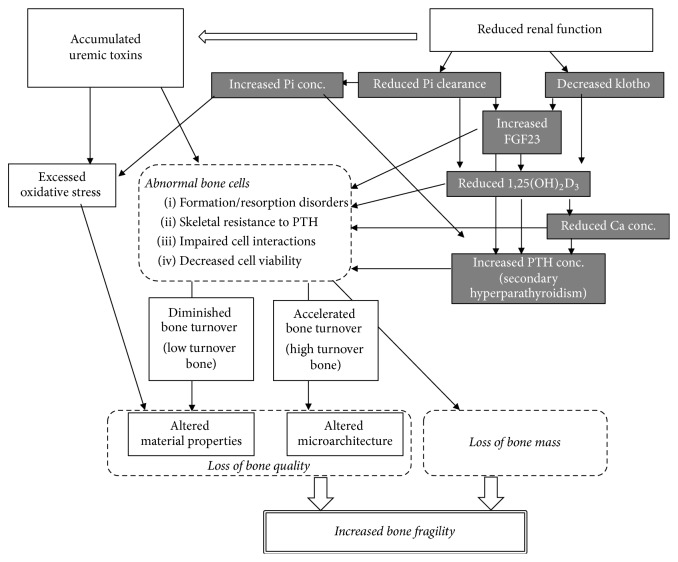
Possible factors involved in bone fragility. Both mineral metabolism disorders and uremic condition induce bone fragility. The detailed mechanisms and interactions are described in the text. Gray-shaded boxes indicate the phenomena induced by mineral metabolism disorders. Detailed descriptions of components of bone quality are shown in Table 1. Pi, phosphorus; Ca, calcium; FGF23, fibroblast growth factor 23; conc, concentration.

To summarize, bone fragility in CKD is probably caused by loss of bone mass and deterioration of bone quality through changes in blood levels of humoral factors and the presence of uremic toxins (Figure 1).

## 8. Pharmacological Therapeutics for Bone Fractures in CKD Patients

In the general population, pharmacotherapy is the mainstay of management for osteoporosis. Patients with primary osteoporosis are treated with different types of drugs. Guidelines for primary osteoporosis recommend antiresorptive drugs (bisphosphonates, antagonists of osteoclasts, and selective estrogen receptor modulators) and stimulators of bone formation (teriparatide) as well as active vitamin D and calcium supplementation. However, these drugs present problems for CKD patients, because some are excreted via the kidneys. The KDIGO guidelines [30] indicate that extrapolating results from studies of osteoporosis in general population to patients with CKD stages 3–5D may not be valid, with concerns over long-term safety because the pathogenesis differs between primary osteoporosis and CKD-MBD-related osteoporosis. On the other hand, due to the increases in osteoporosis and CKD with advancing age and the proven safety profile of osteoporotic agents, the KDIGO guidelines approve the use of these agents in early CKD patients with high risk of fracture, including patients with osteoporosis and CKD stages 1-2. Potential treatments with antiosteoporotic agents in different CKD stages are summarized in Table 3. Additional information for some agents will be discussed in detail below.

Although bisphosphonates have become a standard treatment for osteoporosis, bisphosphonates should not be used in patients with stages 4-5 CKD because these drugs are excreted by the kidney. The accumulation of bisphosphonates in bone also needs to be considered. Ott [106] reported the accumulation of bisphosphonate in the bone of dialysis patients treated with these agents. The use of bisphosphonate in dialysis is a growing concern, as there is the possibility of causing “frozen bone” with extremely low bone turnover. Bisphosphonate exposure over a 5.5-year period was reported to aggravate bone viscoelasticity and provoke atypical femoral fractures [107]. This phenomenon may be a consequence of reduced heterogeneity of material properties through suppressed bone turnover [108]. Use of bisphosphonates may increase the fracture risk through exacerbation of bone mechanical properties and increase atypical fractures [109–111]. Since the degree of bisphosphonate accumulation and the efficacy of bisphosphonates both depend on their affinity to hydroxyapatite, existing data suggest treatment durations of up to 5 years with alendronate, 3 years with zoledronate, and 1 year with risedronate, although the optimal length of a “drug holiday” has not been established [112]. If it is necessary to use bisphosphonates for the management of severe osteoporosis in patients with CKD, bisphosphonates that have low affinity to hydroxyapatite crystals, such as risedronate and ibandronate, should be chosen.

Denosumab, a human monoclonal antibody for the receptor activator of nuclear factor-kappa B, does not accumulate in the body because its point of action is limited. Its efficacy in CKD is expected to be the same as that in primary osteoporosis. A previous study reported that the efficacy of denosumab, which increases BMD and suppresses fractures, did not differ depending on kidney function [113]. Another study reported that denosumab significantly increased BMD of the lumber spine and femoral neck in hemodialysis patients, although the sample size was small [114]. Denosumab may induce hypocalcemia through strong suppression of bone resorption, which tends to be amplified in CKD patients [115]. Denosumab should be prescribed with active vitamin D to regulate the calcium balance.

Raloxifene, a selective estrogen receptor modulator improved BMD in postmenopausal women with CKD, and greater increases in BMD were associated with lower creatinine clearance [116]. In another study, patients on raloxifene showed slower progression of kidney disorders and significantly fewer kidney-related adverse events compared to the placebo group [117]. However, reduced serum calcium concentration and increased PTH secretion were reported.

Teriparatide is a recombinant protein of PTH (1–34) and an anabolic agent for the treatment of postmenopausal osteoporosis. Although teriparatide should be used with caution in osteoporotic patients with CKD due to higher blood PTH level in secondary hyperparathyroidism associated with CKD, intermittent PTH administration can be used to induce an anabolic effect on bone in CKD. Some studies have reported that teriparatide treatment increases BMD [118–120] and ameliorates bone turnover [120]. Subjects of these studies showed decreased endogenous PTH concentration compared to appropriate controls. The effect of teriparatide treatment on CKD patients with normal or slightly higher PTH remains unknown.

Anti-sclerostin monoclonal humanized antibodies such as romosozumab and blosozumab, a new class of drugs with novel mechanisms of action, are being developed for osteoporosis treatment. In clinical trials, romosozumab and blosozumab have been shown to increase bone mass concomitant with increase in bone formation marker and decreases in bone resorption markers [121, 122]. Increases not only in trabecular BMD but also in cortical thickness and stiffness assessed by HR-pQCT were observed in subjects taking romosozumab compared to placebo controls [123]. Although elevated levels of sclerostin have been reported in CKD stages 3 to 5D patients [73, 124, 125], there are no clinical data on anti-sclerostin antibody treatment in CKD patients. In phase 2 in clinical trial of romosozumab, subjects who had estimated creatinine clearance as low as 30 mL/min were included [121]. Since romosozumab was associated with favorable effects on bone turnover in that study population, its efficacy in improving bone fragility in CKD patients may be anticipated.

In addition to the apparent relationship between sclerostin and bone strength, blood level of sclerostin has been shown to be associated with aorta valve calcification [126] and cardiovascular mortality in CKD patients [127, 128]. Further studies are needed to investigate the efficacy of sclerostin antibody treatment not only for fracture prevention but also for reducing cardiovascular mortality in CKD patients.

Control of hyperphosphatemia is important for CKD patients to prevent cardiovascular events and reduce the risk of death. From a secondary analysis of the EVOLVE trial, cinacalcet reduced the rate of clinical fractures by 16–29% [129]. The BONAFIDE trial demonstrated that long-term treatment with cinacalcet substantially reduced PTH and diminished elevated bone turnover as well as several biomarkers [130]. Yamamoto et al. [131] reported that dialysis patients who received angiotensin-converting enzyme inhibitors or angiotensin II type I receptor blockers had an approximately 30% lower risk of hospitalization for any fracture. It is possible that, in addition to traditional antiosteoporotic drugs, the use of inhibitors of specific pathophysiological conditions associated with renal failure is an appropriate strategy for the treatment of osteoporosis in CKD.

## 9. Conclusion

Determining the pathogenesis of osteoporosis and treatment efficacy is difficult in CKD patients because of the complicated mineral and bone abnormalities in these patients. As described above, many factors such as BMD, humoral factors, and alterations of material properties potentially affect bone strength. However, the factors that contribute to bone strength in the setting of CKD and their mechanisms of action remain unknown. For example, no changes in structural parameters and bone mechanical parameters were observed 6 months after kidney transplantation, even though BMD was ameliorated [132]. Other report revealed that cortical porosity is not superior to BMD determined by DXA hofr identification of HD patients with fragility fracture [133]. Moreover, clinical assessment of human femoral mechanical properties by reference point indentation (RPI), which is a novel technique that allows direct measurement of bone material or biomechanical properties, indicated that BMD did not discriminate fracture cases form controls [134]. These recent studies suggest that bone strength in CKD patients may be affected by many factors in a complicated manner, and the major factor and its degree of contribution remain unidentified. Therefore, more studies are required to assess bone mechanical properties using a multitude of factors including BMD and humoral factors. If PRI can be used easily in clinical studies, we may be able to discuss the diagnosis or grading of bone fragility in CKD patients. CKD patients are at increased risk for fractures regardless of whether they are on dialysis. The KDIGO working group is scheduling a selective revision of the guidelines [135]. However, until then, patients at risk of fragility fractures still need to be managed. Researchers, clinicians, pharmacologists, nurses, drug companies, and other authorities should pay particular attention to osteoporosis in CKD patients to determine suitable management.

## Figures and Tables

**Table 1:** Components of bone quality.

Structural properties		Material properties	
Analytical method	Parameter	Analytical method	Parameter
Bone histomorphometry	Trabecular number	Bone histomorphometry	Mineralization
	Trabecular thickness		
	Trabecular connectivity	FTIR, Raman	Relative mineralization
	Cortical thickness		Collagen crosslinking ration
			Crystal size, purity, perfection
MicroCT, pQCT, HR-pQCT	Cortical thickness	HPLC	Collagen crosslinking type
	Cortical porosity		
	Trabecular number	Back scattered electron imaging	Mineral density distribution
	Trabecular thickness		
	Trabecular connectivity	EDX	Elemental analysis
Bone histomorphometry, CMS	Microdamage length, density	X-ray diffraction	Apatite orientation

Micro-CT, microcomputed tomography; pQCT, peripheral quantitative computed tomography; HR-pQCT, high-resolution peripheral quantitative computed tomography; CMS, contact microradiograph; FTIR, Fourier transform infrared spectroscopy; HPLC, high-performance liquid chromatography; EDX, energy-dispersive X-ray spectroscopy.

**Table 2:** Molecular abnormalities that affect bone loss and bone quality.

Category	Factor	Loss of bone mass	Deterioration of bone quality
Humoral factors	PTH	Activating bone resorption and modulating bone turnover [42, 43, 46–50]	Modulating microarchitecture [41]
	FGF23	Inhibiting bone formation [64]	Inhibiting mineralization [65]
	Sclerostin	Inhibiting bone formation [66–68, 70, 71, 75]	Inhibiting mineralization [71]
			Modulating material property [74]
	Vitamin D	Inhibiting bone formation [76, 77]	Inhibiting mineralization [76]
Uremia-specific	Uremic toxins and advanced oxidative stress	Modulating bone turnover [92–97]	Modulating material property [51, 83–88]
Bone aspects	Microcrack accumulation, osteocytes apoptosis		Modulating material property [101–103]

Numerals are reference numbers.

**Table 3:** Pharmacotherapies for osteoporosis according to stage of chronic kidney disease (CKD).

Agents	CKD stage ≤ 3 without biochemical abnormalities	CKD stage > 3 with biochemical abnormalities	Dialysis (stage 5D)
Alendronate	+	−	+
Risedronate	+	−	−
Etidronate	−	−	−
Ibandronate	+	+	+
Minodronate	+	+	+
Denosumab	+	+	+
Raloxifene	+	+	+
Teriparatide	+	+	+

+: use with caution; −: avoid use.
